# The emerging role of the HTRA1 protease in brain microvascular disease

**DOI:** 10.3389/frdem.2023.1146055

**Published:** 2023-04-12

**Authors:** Christof Haffner

**Affiliations:** Department of Psychiatry and Psychotherapy, School of Medicine, Klinikum rechts der Isar, Technical University of Munich, Munich, Germany

**Keywords:** HTRA1, microvascular disease, small-vessel disease, CADASIL, CARASIL, cerebral amyloid angiopathy, proteomics

## Abstract

Pathologies of the brain microvasculature, often referred to as cerebral small-vessel disease, are important contributors to vascular dementia, the second most common form of dementia in aging societies. In addition to their role in acute ischemic and hemorrhagic stroke, they have emerged as major cause of age-related cognitive decline in asymptomatic individuals. A central histological finding in these pathologies is the disruption of the vessel architecture including thickening of the vessel wall, narrowing of the vessel lumen and massive expansion of the mural extracellular matrix. The underlying molecular mechanisms are largely unknown, but from the investigation of several disease forms with defined etiology, high temperature requirement protein A1 (HTRA1), a secreted serine protease degrading primarily matrisomal substrates, has emerged as critical factor and potential therapeutic target. A genetically induced loss of HTRA1 function in humans is associated with cerebral autosomal-recessive arteriopathy with subcortical infarcts and leukoencephalopathy (CARASIL), a rare, hereditary form of brain microvascular disease. Recently, proteomic studies on cerebral amyloid angiopathy (CAA), a common cause of age-related dementia, and cerebral autosomal-dominant arteriopathy with subcortical infarcts and leukoencephalopathy (CADASIL), the most prevalent monogenic small-vessel disease, have provided evidence for an impairment of HTRA1 activity through sequestration into pathological protein deposits, suggesting an alternative mechanism of HTRA1 inactivation and expanding the range of diseases with HTRA1 involvement. Further investigations of the mechanisms of HTRA1 regulation in the brain microvasculature might spawn novel strategies for the treatment of small-vessel pathologies.

## Introduction

Brain microvascular disease is a major cause of vascular dementia and invariably associated with magnetic resonance imaging (MRI) abnormalities such as white matter hyperintensities, lacunes (small fluid-filled cavities), microbleeds, enlarged perivascular spaces, or cortical superficial siderosis (Wardlaw et al., 2019). These abnormalities do not necessarily manifest in acute clinical symptoms such as ischemic or hemorrhagic stroke, but cumulatively also contribute to cognitive impairment in a subacute manner. Due to their high prevalence in elderly individuals, cerebral small-vessel disease (SVD) has emerged as major illness of the aging brain. It represents a heterogeneous group of conditions including cerebral amyloid angiopathy (CAA) and non-amyloid arteriopathies such as arteriolosclerosis, also known as hypertensive or age-dependent SVD (Pantoni, 2010; Charidimou et al., 2017). In addition to these common, sporadic disorders, several rare, hereditary SVD forms linked to monogenic causes are known, most importantly cerebral autosomal-dominant arteriopathy with subcortical infarcts and leukoencephalopathy (CADASIL), cerebral autosomal-recessive arteriopathy with subcortical infarcts and leukoencephalopathy (CARASIL), hereditary cerebral hemorrhage with amyloidosis-Dutch type (HCHWA-D), and COL4A1/A2-associated arteriopathies (Haffner et al., 2016; Biffi, 2022). Brain microvascular pathologies affect vessels of different size and location within the cerebrovascular tree: small pial arteries and arterioles at the brain surface, penetrating arterioles, cortical and subcortial capillaries and venules. Depending on their diameter, vessels have different wall architectures, varying in thickness and mural cell composition: while arterioles encompass up to several layers of contractile smooth muscle cells (SMCs) surrounded by extracellular matrix (ECM), capillaries contain only isolated pericytes embedded into the endothelial basement membrane, a specialized ECM layer (Schaeffer and Iadecola, 2021). A recurrent feature of microvascular pathologies is the severe disruption of this architecture manifesting in ECM remodeling, basement membrane thickening or mural cell degeneration (Fang et al., 2022). The molecular processes underlying these alterations are poorly understood, but some insight has been gained from recent studies on disease forms with defined etiology.

## Molecular etiologies of microvascular disease

The aggregation and cerebrovascular deposition of amyloid-β (Aβ) peptides, the products of sequential processing of the amyloid precursor protein (APP), in the walls of medium-sized and small brain vessels is the defining feature of CAA, a major cause of intracerebral hemorrhage (Charidimou et al., 2017; Greenberg et al., 2020). CAA is a primarily sporadic condition frequently detected in autopsy tissue of elderly individuals, particularly Alzheimer's disease (AD) patients, and thus considered as a common contributor to age-related dementia (Greenberg et al., 2020). The causal role of Aβ in CAA development is corroborated by the existence of hereditary forms associated with mutations in the APP gene, most prominently APP^E693Q^ linked to HCHWA-D (Biffi, 2022). Aβ deposits are typically detected in leptomeningeal and cortical vessels and can occur either with or without the involvement of capillaries resulting in CAA type-1 (also known as capillary CAA) or CAA type-2. Type-1 has been reported to contribute to a large extent to memory decline by promoting hippocampal microinfarcts (Hecht et al., 2018). CAA is widely believed to largely develop due to incomplete clearance of Aβ from the brain parenchyma, the primary location of its production (Greenberg et al., 2020; Zhao et al., 2022). In addition to intracerebral metabolism and efflux across the blood-brain barrier, perivascular pathways have emerged as important Aβ drainage routes. A glymphatic pathway involving extramural fluid flow within the perivascular space as well as an intramural periarterial drainage (IPAD) route along the basement membranes of capillaries and arteries were described (Carare et al., 2008; Iliff et al., 2012), but their relative importance for Aβ clearance is a matter of debate (Zhao et al., 2022). In any case, the age-dependent reduction of perivascular drainage system capacities is likely to trigger Aβ deposition in the cerebrovasculature, thus initiating pathological processes such as extracellular matrix remodeling, basement membrane thickening and mural cell loss (Howe et al., 2020). Despite the clear link between these alterations and vessel rupture and bleedings, both frequent incidents associated with CAA, a deep understanding of the involved molecular pathomechanisms is lacking.

Excessive vascular protein deposition is also a hallmark of CADASIL, the most common monogenic inherited SVD, which predominantly manifests in ischemic stroke and vascular dementia (Chabriat et al., 2009). Although much less frequent than CAA, CADASIL has become a well-studied cerebrovascular model disease. Characteristic deposits in vessel walls show an amorphous, non-amyloid structure and are detectable by electron microscopy as granular osmiophilic material (GOM) (Tikka et al., 2014). The primary GOM constituent is the extracellular domain of the Notch3 receptor (Notch3^ECD^), which is expressed at the surface of mural cells and activated by membrane-bound ligands on neighboring cells (Del Gaudio et al., 2022). While Notch3 signaling is required for vessel integrity and mural cell function (Schoemaker and Arboleda-Velasquez, 2021), CADASIL is widely believed to be caused by a neomorphic mechanism based on Notch3^ECD^ aggregation and accumulation (Joutel et al., 2016; Haffner, 2019; Gravesteijn et al., 2022). Pathogenic mutations locate to the Notch3^ECD^ and typically alter the number of cysteine residues in one of the tandemly arrayed epidermal growth factor (EGF) domains (Chabriat et al., 2009; Rutten et al., 2018). This results in a disruption of the conserved disulfide bond pattern and in local Notch3^ECD^ misfolding which, however, rarely interferes with Notch3 signaling. Misfolded Notch3^ECD^ is likely to resist clearing from the extracellular space and to trigger an aggregation process eventually leading to the formation of large, insoluble deposits (Haffner et al., 2016; Joutel et al., 2016). The subsequent recruitment of additional proteins is believed to represent a crucial step in disease pathogenesis. Although some progress has been made in identifying downstream processes promoting vascular dysfunction (Capone et al., 2016; Ehret et al., 2021; Oka et al., 2022), many molecular details are still unknown.

The rare, recessively inherited arteriopathy CARASIL shares several features with CADASIL including early-onset ischemic stroke, subcortical MRI abnormalities and a microvessel pathology in the absence of vascular risk factors such as hypertension (Nozaki et al., 2014). Neuropathological characteristics include severe arteriolosclerosis, loss of smooth muscle cells, and splitting of the internal elastic lamina (Oide et al., 2008). The classification of CARASIL as a distinct disease entity was initially based on its recessive inheritance pattern, the lack of vascular protein deposits and the frequent presence of extracerebral symptoms such as alopecia and lumbago. A seminal genetic linkage study indeed identified mutations in a gene—high temperature requirement protein A1 (HTRA1)—previously not linked to vascular disease (Hara et al., 2009). HTRA1 encodes an evolutionary conserved serine protease with a broad substrate specificity and a unique mode of activation (Clausen et al., 2011). Mutations result in an impairment of HTRA1 activity and occur homozygously in CARASIL patients, in agreement with a loss-of-function mechanism (Nozaki et al., 2016). However, also heterozygous HTRA1 mutations were found to be pathogenic and to cause a dominantly inherited, less severe small-vessel arteriopathy (Verdura et al., 2015). Both haploinsufficiency and a dominant effect interfering with HTRA1 activation have been suggested as underlying mechanism (Uemura et al., 2019; Coste et al., 2021). Meanwhile, HTRA1 mutations are considered the second most common cause of hereditary SVD (Cho et al., 2022) and both rare and common variants have been linked to white matter pathologies in the general population (Mishra et al., 2019; Malik et al., 2021). Additionally, recent proteomic studies in CAA and CADASIL have provided evidence for impaired HTRA1 activity suggesting a role in diseases not caused by HTRA1 mutations (Zellner et al., 2018, 2022).

## High temperature requirement protein A1

The family of trypsin-like HTRA serine proteases is conserved in both unicellular and multicellular organisms and primarily involved in various aspects of protein quality control (Clausen et al., 2011). In many species, HTRA proteases contribute to the cellular response to protein folding stress by degrading misfolded and mislocalized proteins in the extracellular space. Their catalytically active unit is a trimer or a higher multimer which undergoes reversible activation through an allosteric mechanism involving an intramolecular signal relay system (Clausen et al., 2011; Cabrera et al., 2017). HTRA1, one of four human family members, is a primarily secreted protein originally linked to tumorigenesis and arthritis and subsequently implicated in other conditions including age-related macular degeneration (AMD) (Clausen et al., 2011). Its major function in the human organism remained unclear until the identification of the association of genetic mutations with hereditary cerebral arteriopathies (see above). Mutations result in a loss of HTRA1 activity by several mechanisms including structural alteration of the catalytic domain, reduction in mRNA levels through nonsense-mediated mRNA decay and HTRA1 protein truncation. Structural mutations either prevent trimerization or interfere with the intramolecular activation mechanism (Uemura et al., 2019). HTRA1 has a broad substrate specificity cleaving predominantly extracellular proteins (An et al., 2010; Zellner et al., 2018), however, the *in vivo* relevance of most substrates is unclear. The transforming growth factor-β (TGFβ) pathway was repeatedly reported to be dysregulated by mutant HTRA1, but data are conflicting with regard to pathway activation or inhibition (Hara et al., 2009; Beaufort et al., 2014; Chuanfen et al., 2022). Recently, the brain microvessel proteome of HTRA1-deficient mice was determined establishing a loss-of-function profile (Zellner et al., 2018; Kato et al., 2021). While this did not resolve the debate on the TGFβ pathway, it considerably broadened our knowledge about matrisomal substrates such as fibronectin, vitronectin and clusterin. A similar analysis in patients is currently not possible due to the lack of appropriate *post mortem* tissue. However, proteomic analyses of vessel samples derived from CAA and CADASIL patients revealed an unexpected link to HTRA1 providing further insight into its role in humans (Zellner et al., 2018, 2022).

## Role of HTRA1 in Aβ and Notch3^*ECD*^ deposition

Initial evidence for an involvement of HTRA1 in amyloid aggregation diseases was presented in biochemical *in vitro* studies showing HTRA1-dependent degradation of Aβ and tau, the principal components of AD-associated amyloid plaques and neurofibrillary tangles (Grau et al., 2005; Tennstaedt et al., 2012; Poepsel et al., 2015). Moreover, also solubilization and disintegration of Aβ aggregates and tau fibrils were observed, in principle allowing productive HTRA1-mediated degradation of pathological deposits. The colocalization of HTRA1 with parenchymal Aβ plaques in patient brain tissue sections further strengthened this link (Grau et al., 2005), but additional data from humans did not emerge until recently, when elevated HTRA1 levels were reported in two proteomic studies investigating AD brain samples (Bai et al., 2020; Drummond et al., 2022). The first evidence for a role in vascular Aβ pathology came from a proteomic study in Tg-SwDI mice, the most widely used rodent CAA model carrying a human APP transgene with the APP^K670N/M671L^ (“Swedish”) mutation and the two vasculotropic APP^E693Q^ (“Dutch”) and APP^D694N^ (“Iowa”) mutations (Searcy et al., 2014). HTRA1 was identified as the protein with the strongest increase in abundancy during the age-dependent advancement of Aβ pathology. A similar result was recently obtained in rTg-DI rats, a novel model for CAA type-1 (Schrader et al., 2022).

The improvement of techniques for brain vessel isolation spurred approaches to determine the microvessel proteomes in SVD patients, most importantly CAA and CADASIL (reviewed in Haffner, 2019). Initial attempts suffered from insufficient proteome depth and high sample heterogeneity yielding reliable data on HTRA1 in only one study (Hondius et al., 2018). Using laser-microdissected vascular tissue samples, HTRA1 could be detected only in CAA type-1, but not in control samples preventing quantification and the precise determination of abundance alterations. Immunohistochemistry revealed an intense HTRA1 staining of arteriolar walls in patient samples, suggesting accumulation in vessels with Aβ pathology. A very recent proteomic study applying an advanced microvessel isolation technique to 10 CAA type-1 patients (including a HCHWA-D case) and 11 control individuals provided sufficient depth for a comprehensive analysis (Zellner et al., 2022). Tandem liquid chromatography mass spectrometry (LC-MS/MS) followed by label-free quantification resulted in the quantification of >3,750 proteins and identified HTRA1 (4.7-fold increase) as one of the most highly enriched proteins in the patient group. Immunofluorescence staining of isolated capillaries followed by high-resolution confocal microscopy demonstrated a segmental distribution highly similar to Aβ and an almost perfect colocalization. This strongly suggested recruitment of HTRA1 to microvascular Aβ deposits.

As in CAA, proteomic approaches applied to CADASIL patient samples initially provided incomplete data for a role of HTRA1, mainly due to low sample numbers (Haffner, 2019; Young et al., 2020). In a pilot study on two brain samples enriched for insoluble Notch3^ECD^, HTRA1 was detected in the patient, but not in the control sample (Monet-Lepretre et al., 2013). While this suggested accumulation in CADASIL-affected vessels, neither a quantification nor a statistical analysis could be conducted. The so far most comprehensive proteomic analysis of CADASIL samples was performed on six patients carrying five different Notch3 mutations and six healthy controls (Zellner et al., 2018). An advanced method for the preparation of microvessels from brain samples was combined with high-resolution LC-MS/MS and label-free quantification yielding a proteomic CADASIL profile of unprecedented depth and revealing a 4.9-fold HTRA1 accumulation. Immunoblotting experiments on vessel lysates confirmed this finding, and confocal immunofluorescence microscopy on isolated microvessels demonstrated a high degree of colocalization between HTRA1 and Notch3^ECD^ deposits indicating a recruitment process.

The shared set of accumulating proteins - most importantly serum amyloid P component (SAP, encoded by the APCS gene), apolipoprotein E (APOE), vitronectin and clusterin - consistently observed in the initial CAA and CADASIL proteome studies indicated common pathological processes resulting in microvascular dysfunction (Haffner, 2019; Young et al., 2020). Suggested scenarios included increased protein secretion, decreased protein turnover through formation of stable protein complexes, enhanced protein aggregation due to intrinsic metastability and recruitment of proteins with chaperone activity. A meaningful evaluation of the role of HTRA1 became possible through a comparison of the most recent, high-depth CAA and CADASIL profiles with the HTRA1 loss-of-function signature from HTRA1-deficient mice.

## Mechanisms of HTRA1 inactivation

**Figure 1:**
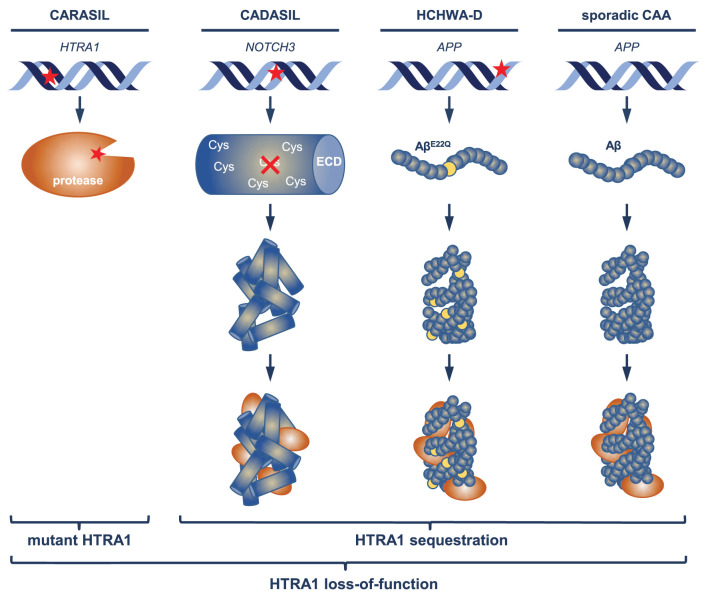
Mechanisms of HTRA1 inactivation. In CARASIL patients, enzymatic activity of HTRA1 is primarily impaired by mutations (indicated by the red asterisk) altering the structure of the protease domain. Mutations causing CADASIL and HCHWA-D occur in NOTCH3 (affecting cysteine residues) respectively APP (APP^E693Q^) and result in the aggregation of mutant Notch3^ECD^ and Aβ (Aβ^E22Q^); in sporadic CAA, aggregates consist of wild-type Aβ. Recruitment of HTRA1 to pathological deposits may lead to its sequestration and depletion in the extracellular space.

A large fraction of the set of accumulating proteins shared between the CAA and CADASIL proteome profiles also overlapped with the HTRA1^−/−^ mouse profile suggesting the existence of a HTRA1 loss-of-function signature (Zellner et al., 2018, 2022; Kato et al., 2021). Evidence for an impairment of HTRA1-mediated proteolytic activity in the cerebrovasculature has so far only been found in patients carrying congenital mutations (Nozaki et al., 2014), thus HTRA1 inactivation via sequestration on a protein level would represent a novel pathomechanism (Figure 1). CAA and CADASIL pathogenesis is clearly initiated by the aggregation of misfolded Aβ and Notch3^ECD^, respectively, but a significant fraction of the pathological events resulting in vessel dysfunction might be promoted by a loss of HTRA1-mediated protein clearing activity. In an effort to remove misfolded and aggregated proteins, HTRA1 might be recruited to pathological deposits, with the possible consequence of sequestration and depletion from its normal location. This could lead to a vicious cycle including aggravation of disrupted vascular protein homeostasis, progressive protein accumulation and permanent impairment of HTRA1 activity. The lack of detectable protein deposits in HTRA1-associated microangiopathies suggests that excessive focal protein accumulation is a cause and not a consequence of HTRA1 inactivation.

For the vast majority of proteins constituting the HTRA1 loss-of-function signature, a direct cleavage has been demonstrated *in vitro*. Proteolysis of APOE, whose ε4 allele is a strong risk factor for both CAA and AD, has been shown in cell-based assays and a preferential cleavage of ApoE-ε4 was observed (Chu et al., 2016). Furthermore, HTRA1-mediated ApoE fragmentation by HTRA1 was reported to generate a fragment with neurotrophic properties (Munoz et al., 2018). Clusterin, a chaperone involved in CAA pathogenesis (Endo et al., 2019), was shown to be degraded by HTRA1 in an *in vitro* assay using purified proteins (An et al., 2010). Likewise, direct cleavage of matrisomal proteins including tissue inhibitor of metalloprotease 3 (TIMP3) and vitronectin was demonstrated in cell-based assays (Zellner et al., 2018). The lack of HTRA1-mediated substrate processing or degradation is thus likely to contribute to the deterioration of vessel structure and function in microvascular disease.

## Summary and outlook

The identification of a link between several distinct SVD forms, both hereditary and sporadic, and the HTRA1 protease suggests the existence of at least partially shared pathomechanisms underlying brain microvascular disease. The disruption of protein homeostasis in the vessel wall, triggered by reduced HTRA1 activity, might represent a crucial step in the development of small-vessel pathologies. Remarkably, persistent HTRA1 inactivation does not only take place on DNA level as a result of congenital mutations, it is also likely to occur on protein level through sequestration of HTRA1 into pathological aggregates. An enhancement of HTRA1 activity may thus represent a promising strategy to counteract the detrimental processes associated with SVD. A similar approach might also be useful in other conditions with HTRA1 involvement such as neurodegenerative diseases (Shorter, 2017), age-related macular degeneration (Gerhardy et al., 2022) and TGFβ-induced protein (TGFBIp)-linked corneal dystrophies (Nielsen et al., 2020). Of note, there is only limited evidence for a transcriptional regulation of HTRA1 in dementia disorders. Single-cell transcriptome analysis of brain vessel preparations from AD/CAA patients failed to detect differential HTRA1 expression in cerebrovascular cell types (Yang et al., 2022). In a similar study on cortical AD samples and major brain cell types, no HTRA1 transcript level differences were measured with the exception of oligodendrocytes, where a moderate increase was observed (Mathys et al., 2019). In contrast, proteomic studies on microglia isolated from several AD mouse models reported strongly increased HTRA1 protein levels indicating a role in the immunological response to the disruption of cerebral protein homeostasis (Rangaraju et al., 2018; Monasor et al., 2020). Thus, further exploring the details of the regulation of HTRA1 activity will provide valuable insights into brain disease pathomechanisms and might lead to novel treatment strategies.

## Author contributions

CH wrote the manuscript and prepared the figure.
